# The BB Wistar Rat as a Diabetic Model for Fracture Healing

**DOI:** 10.1155/2013/349604

**Published:** 2013-03-31

**Authors:** Amit Sood, Catie Cunningham, Sheldon Lin

**Affiliations:** Department of Orthopedic Surgery, University of Medicine and Dentistry of New Jersey, New Jersey Medical School, Newark, NJ 07103, USA

## Abstract

The advent of improved glucose control with insulin and oral medications has allowed for the diabetic population to live longer and healthier lives. Unfortunately diabetes remains a worldwide epidemic with multiple health implications. Specifically, its affects upon fracture healing have been well studied and shown to have negative effects on bone mineral density, biomechanical integrity, and fracture healing. Multiple animal models have been used for research purposes to gain further insight into the effects and potential treatments of this disease process. The diabetic BB Wistar rat is one model that replicates a close homology to human type-1 diabetes and has been used as a fracture model to study the effects of diabetes on bone integrity and healing. In particular, the effects of tight glucose control, ultrasound therapy, platelet-rich plasma (PRP), platelet-derived growth factor (PDGF), bone morphogenetic protein 2 (BMP-2), and allograft bone incorporation have been studied extensively. We present a review of the literature using the BB Wistar rat to elucidate the implications of diabetes on fracture healing.

## 1. Clinical Significance

In the United States, over 13 million Americans have been diagnosed with diabetes mellitus (DM) and an estimated 40 million Americans will develop DM over the next 10 years [1]. The advent of the improved insulin regiment and/or oral hypoglycemics has led to a DM population that is more active and ultimately lives longer. Unfortunately, treatment of DM fractures presents a challenge to the orthopaedic surgeon. Several clinical series, analyzing fracture healing in patients with DM, demonstrated a significant incidence of delayed union, nonunion, and pseudarthrosis [2–5]. Diabetic osteopathy, as one of the diabetes-induced complications, leads to diminished bone formation [6], retardation of bone healing [2], and osteoporosis [7–9]. Bone mineral density [10] and biomechanical integrity [3, 11] are referential predictors of fracture, and patients with type-1 diabetes (T1D) also incur a higher incidence of fractures than healthy individuals. In addition to altered biomechanical properties, diabetic fracture callus has shown to have reduced cell proliferation and collagen synthesis during early states of fracture healing [2, 12, 13]. Patients with T1D are particularly vulnerable to hip fracture [14]. Women with T1D have a 6.9 to 12-fold likelihood of hip fractures compared to women without DM [15, 16]. Fracture healing in patients with all forms of DM may also take twice as long as nondiabetic patients [5, 17]. Likewise, these patients sustain compromised fracture healing.

Clinical statistics for other types of fractures are equally sobering. Patients with DM treated operatively for ankle fracture are likely to have worse results than nondiabetic patients with regard to postoperative complications, length of hospital stay, and mortality [18]. Moreover, ankle fractures in patients with DM lead to an increased rate of infection due to peripheral vascular disease [19]. In addition, diabetic patients who incur fractures in bones of their feet can have serious problematic sequelae. Low bone mineral density in these individuals, exacerbated by casting, can lead to fracture recurrence and progression to foot deformity [20]. Furthermore, a complication rate of 42-43% exists in diabetic patients following surgery to treat ankle fractures compared to a 0–16% complication rate in control patients [21, 22]. Complications include reduction in callus bone content, malunion, infection, revisions, and amputation in some cases. This was underscored by White et al. who reported patients with T1D had statistically higher risk for amputation after open ankle fractures (75%) compared to patients with type II DM (10%) and nondiabetic patients (3%) [23].

## 2. BB Wistar Rat Model

Our laboratory has investigated fracture healing in the DM BB Wistar rat, a laboratory animal that represents a close homology of human T1D [24, 25]. The onset of DM in BB Wistar rats is spontaneous which confers advantages over the viral, chemical, and immunological induction of DM. The BB Wistar rat develops DM through an autoimmune process with selective destruction of the pancreatic beta cells (intense insulintis). As a result, within 7 days of the onset of glycosuria, the beta cells are completely destroyed and if untreated, marked body wasting (including fat and muscle tissue), dehydration, and ketosis supervene. Death usually results within 5–10 days after onset. Such conditions, however, are resolved with insulin treatment. 

In contrast, STZ or alloxan-induced DM models consist of selective poisoning of pancreatic beta cells. Alloxan was the initial agent but has been replaced by STZ as the primary diabetogen for experimental DM. Alloxan and STZ are thought to cause DNA strand breaks which activate the repair mechanism nuclear poly(ADP-ribose) synthetase and deplete the cellular pool of NAD+, resulting in pancreatic P-cell damage [26, 27].

Based upon a previous investigation performed within our laboratory using BB Wistar rats [25], one group of DM animals, denoted TC (tightly controlled), was maintained with blood glucose (BG) levels <170 mg/dL and demonstrated a well-controlled state of DM exhibiting no signs of glycosuria or ketonuria. Another group of DM animals, denoted LC (loosely controlled), was maintained with BG levels >300 mg/dL and demonstrated a poorly controlled state of DM with glycosuria but no sign of ketonuria. Blood specimens may be obtained from tail veins and tested for blood glucose levels. Insulin implants (LINPLANT) may be aseptically placed subcutaneously in the dorsal neck which provides constant insulin release for approximately 30 days. If the desired BG level is not achieved, additional insulin implants may be given to achieve the appropriate level. 

## 3. Adjunct Treatments for Diabetic Fracture Healing

A number of studies have been published using the BB Wistar rat as a diabetic model. This section highlights the studies which have attempted to describe the clinical impact of DM on bone healing.

### 3.1. Blood Glucose and Fracture Healing

**Figure 1:**
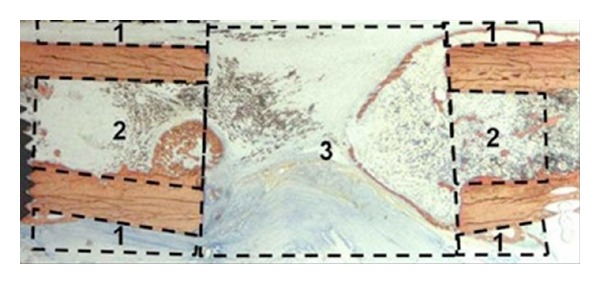
Histological sample of bone illustrating the areas measured within Table 1. Region (1) is periosteal bone, (2) is endosteal bone, and (3) is defect bone [40, 41].

Insulin receptors have been identified in rat osteoblastic cells, and insulin has been shown *in vitro* to stimulate nucleotide synthesis of osteoblasts, proliferation of osteoblastic cells, and to be related to collagen production in fetal rat calvariae and the presence of IGF-1, which stimulates both collagen synthesis and cell proliferation [28–36]. Beam et al. evaluated the effects of insulin and blood glucose (BG) control on fracture healing in the DM BB Wistar rat compared to loosely controlled DM and non-DM rats [25]. This study showed decreased cell proliferation and decreased chondrogenesis in poorly controlled DM rats. In addition, percent mechanical stiffness, torque to failure, and ultimate shear stress in DM animals with physiologic BG control were similar to non-DM controls.  Table 1 provides a summary of the mechanical effects of tight glucose control and other adjuvant therapies (discussed further in this paper) on fracture healing [25, 36–39].  Figure 1 is a histological photograph of bone illustrating the areas measured within Table 1. The histological delay in endochondral ossification and reductions in the amount of callus bone in LC animals were not observed in TC rats. This study suggests insulin treatment with resultant improved BG control will ameliorate the impaired early and late parameters of DM fracture healing.

Gandhi et al. employed a novel intramedullary insulin delivery system in the diabetic BB Wistar femur fracture model to investigate the potential direct effects of insulin on bone healing as opposed to systemic insulin treatment [36]. Insulin was delivered directly to the fracture site using an insulin-palmitic acid implant placed within a hollow rod which was then inserted within the femoral canal of the rat model at the fracture site. Insulin delivery at the fracture site normalized the early (cellular proliferation and chondrogenesis) and late (mineralized tissue, cartilage content, and mechanical strength) parameters of diabetic fracture healing without affecting the systemic parameters of blood glucose.

### 3.2. Ultrasound Therapy

Low-intensity pulsed ultrasound (LIPUS) has been shown to be a successful adjunct to fracture healing as well as reducing the rate of nonunion in humans [42–45]. In addition, animal studies have demonstrated increased mechanical strength and callus size as well as reduced healing times [46, 47]. Gebauer et al. evaluated the effects of LIPUS on mid-diaphyseal femoral fractures in DM BB Wistar rats. Although LIPUS was shown to have a limited effect on the early proliferative phase of fracture healing, its application did result in improved mechanical strength [37]. The topic of LIPUS use in the DM population was further explored by Coords et al., who studied its effects on growth factor expression, cartilage formation, and neovascularization. Using the DM BB Wistar fracture model, LIPUS was shown to increase all three parameters, to the point where the DM group results resembled those of the non-DM group [48].

### 3.3. Platelet-Rich Plasma

Platelet-rich plasma (PRP) is derived from autologous blood with a platelet count up to 5 times the normal physiologic level. Due to the high number of platelets and the linear relationship demonstrated between PDGF, TGF-*β*, IGF-1, and VEGF levels and platelet count, PRP is considered to be a concentrated source of growth factors integral to bone healing [51–53]. Additionally, our lab has shown these factors to be significantly decreased in diabetic rats [25, 54]. Gandhi et al. investigated the effect of PRP treatment in the diabetic BB Wistar femur fracture model. PRP delivery at the fracture site normalized cellular proliferation and chondrogenesis during early fracture healing and improved overall mechanical strength [38]. 

### 3.4. Platelet-Derived Growth Factor

Platelet-derived growth factor (PDGF) increases collagen deposition, initiates differentiation of progenitor cells towards osteoblastic lineages, and stimulates osteopontin expression [55–57]. As previously stated, PDGF levels in diabetic rats significantly decreased when compared to their non-DM counterparts. Al-Zube et al. hypothesized that application of recombinant human PDGF-BB (rhPDGF-BB) directly to femur fracture sites in DM BB Wistar rats would help mitigate the effect of DM on fracture healing [58]. This study found that rhPDGF-BB treatment promoted early cellular proliferation of the callus, resulting in increased bone formation when compared to controls. 

### 3.5. Bone Morphogenic Protein 2

Bone morphogenic proteins (BMPs) are proteins found in bone with osteoinductive properties, with BMP-2 and BMP-7 being the most extensively researched and used [59]. Various clinical studies have been performed to investigate the efficacy of BMP treatment of fracture healing [60–62]. Azad et al. [40] examined the effects of recombinant human bone morphogenic protein 2 (rhBMP-2) in the presence of systemic disease (diabetes) in a segmental femoral defect model BB Wistar rats. Despite the negative effects of DM on bone healing, application of an rhBMP-2-collagen carrier accelerated new bone formation, with outcome parameters comparable to those of non-DM rhBMP-2 studies. 

### 3.6. Allograft Incorporation in DM Model

While autologous bone graft harvesting may be associated with clinical morbidity, the use of allograft has also raised concern regarding issues with graft incorporation, delayed union at the junction site, immune-related inflammatory complications, and potential for transmission of infectious diseases. A systemic disease such as DM further compounds these complications, providing an impetus to provide an alternative process for bone reconstruction. Breitbart et al. analyzed the effect of DM upon allograft incorporation in a segmental rat femoral defect, finding that less mature bone formed in the DM BB Wistar group compared to its non-DM counterpart. However, this study also investigated the result of mesenchymal stem cell (MSC) augmentation of the allograft, which showed significantly more mature bone in the MSC group when compared to the DM with allograft alone [49]. Similarly, Dedania et al. examined the potential role of local insulin application upon allograft incorporation and found that it significantly accelerated new bone formation [50]. Azad et al. also found recombinant bone morphogenetic protein-2 (rhBMP2) to enhance bone formation within a segmental femoral defect model in a DM BB Wistar rat model [40].  Table 2 provides a summary of the effects of MSC, insulin, and rhBMP2 on allograft bone growth compared to controls from previously published studies. 

## 4. Conclusion

DM is one of the most common medical conditions that exist, and its associated medical complications fuel intense research looking into its pathogenesis and effect on the human body. Although a variety of animal models simulating DM and its complications exist in the literature, the BB Wistar rat model represents a close homology of human type I DM and has been the source of extensive research. Specifically, a large amount of research has been performed using this rat model to investigate the effects of DM on fracture healing. Although early basic science and clinical research has shown the negative impacts that uncontrolled glucose and DM may play in fracture healing and regeneration, more research is needed to further clarify its exact role and what may be done to counteract these effects. The BB Wistar rat provides one model which may help advance such research into this disease process.

## Figures and Tables

**Table 1:** Comparison of percent torque to failure and stiffness using a BB Wistar rat femur fracture model treated with the application of various adjuvants. Mechanical testing was performed after 6 weeks of treatment.

Study	Treatment	*n*	% Torque to failure	% Stiffness
Beam et al., 2002 [25]	DM+, loose glucose control (BG > 300 mg/dL)	6	25 ± 10	29 ± 18
Beam et al., 2002 [25]	DM+, tight glucose control (BG < 170 mg/dL)	5	53 ± 13	80 ± 34
Gebauer et al., 2002 [37]	DM+, LIPUS	5	43 ± 8	55 ± 21
Gandhi et al., 2005 [36]	DM+, insulin	5	54 ± 13	80 ± 34
Gandhi et al., 2006 [38]	DM+, PRP	6	51 ± 14	49 ± 24
Al-Zube et al., 2009 [39]	DM+, low dose rhPDGF-BB (22 *μ*g)	7	72 ± 32	93 ± 28
Al-Zube et al., 2009 [39]	DM+, high dose rhPDGF-BB (75 *μ*g)	7	56 ± 32	52 ± 29
Average of controls from cited studies	DM+, no treatment	32	37.6 ± 15.8	35.6 ± 17.8

Loose glucose control: blood glucose levels > 300 mg/dL, tight glucose control: blood glucose levels < 170 mg/dL, DM: diabetes mellitus, LIPUS: low-intensity pulsed ultrasound, PRP: platelet-rich plasma, rhPDGF: recombinant human PDGF-BB.

**Table 2:** Comparison table of histomorphometrical analysis of bone area using a BB Wistar rat femur fracture model treated with the application of different adjuvants.

Study	Treatment	Time point	*n*	Endosteal bone (mm^2^)	Periosteal bone (mm^2^)	Defect bone (mm^2^)	Total bone (mm^2^)
Azad et al., 2009 [40]	DM+, rhBMP2	3 weeks	(*n* = 6)	1.35 ± 0.48	2.50 ± 0.88	4.05 ± 0.33	7.89 ± 1.00^a^
		6 weeks	(*n* = 6)	0.69 ± 0/30	2.64 ± 1.08	3.83 ± 1.73	7.16 ± 2.44^b^
	*Control group *	3 weeks	(*n* = 6)	0.37 ± 0.23	0.73 ± 0.86	0.46 ± 0.46	0.84 ± 0.39
	DM+, (buffer)	6 weeks	(*n* = 6)	0.67 ± 0.29	1.18 ± 1.01	1.16 ± 1.37	3.01 ± 2.06

Breitbart et al., 2010 [49]	DM+, MSC	4 weeks	(*n* = 5)	1.00 ± 0.39^c^	0.59 ± 0.54	1.97 ± 0.74^d^	3.57 ± 0.80^e^
		8 weeks	(*n* = 7)	1.03 ± 0.77	0.46 ± 0.44	3.46 ± 1.28	4.95 ± 1.98
	*Control group *	4 weeks	(*n* = 7)	0.46 ± 0.26	0.25 ± 0.37	0.37 ± 0.33	1.07 ± 0.69
	DM+, DBM	8 weeks	(*n* = 5)	1.04 ± 0.58	0.32 ± 0.26	1.98 ± 0.49	3.34 ± 0.68

Dedania et al., 2011 [50]	DM+, insulin	4 weeks	(*n* = 7)	1.06 ± 0.27^f^	0.29 ± 0.20	0.31 ± 0.19	1.66 ± 0.13
		6 weeks	(*n* = 7)	2.36 ± 1.66^g^	1.24 ± 0.90	1.85 ± 1.03^h^	5.45 ± 3.04^i^
	*Control group *	4 weeks	(*n* = 6)	0.61 ± 0.17	0.25 ± 0.18	0.31 ± 0.46	1.17 ± 0.65
	DM+, palmitic acid blank	6 weeks	(*n* = 8)	0.54 ± 0.42	0.86 ± 0.55	0.84 ± 0.68	2.24 ± 1.27

DM: diabetes mellitus, MSC: mesenchymal stem cells, DBM: demineralized bone matrix.

^a^Represents values statistically higher than DM/buffer at 3 weeks, *P* < 0.001.

^b^Represents values statistically higher than DM/buffer at 6 weeks, *P* = 0.004.

^c^Represents values statistically higher than DM/DBM at 4 weeks, *P* = 0.006.

^d^Represents values statistically higher than DM/DBM at 4 weeks, *P* < 0.001.

^e^Represents values statistically higher than DM/DBM at 4 weeks, *P* < 0.001.

^f^Represents values statistically higher than DM/palmitic acid at 4 weeks, *P* = 0.006.

^g^Represents values statistically higher than DM/palmitic acid at 6 weeks, *P* = 0.010.

^h^Represents values statistically higher than DM/palmitic acid at 6 weeks, *P* = 0.041.

^i^Represents values statistically higher than DM/palmitic acid at 6 weeks, *P* = 0.017.
